# Novel Wide-Working-Temperature NaNO_3_-KNO_3_-Na_2_SO_4_ Molten Salt for Solar Thermal Energy Storage

**DOI:** 10.3390/molecules29102328

**Published:** 2024-05-15

**Authors:** Huaiyou Wang, Jinli Li, Yuan Zhong, Xu Liu, Min Wang

**Affiliations:** 1 Key Laboratory of Green and High-End Utilization of Salt Lake Resources, Qinghai Institute of Salt Lakes, Chinese Academy of Sciences Xining, Xining 810008, China; lijinli@isl.ac.cn (J.L.); zhongyuan@isl.ac.cn (Y.Z.); liuxu22@mails.ucas.ac.cn (X.L.); 2 Qinghai Provincial Key Laboratory of Resources and Chemistry of Salt Lakes, Xining 810008, China

**Keywords:** molten salts, thermal energy storage, wide working temperature, high specific heat capacity, molecular dynamic simulation

## Abstract

A novel ternary eutectic salt, NaNO_3_-KNO_3_-Na_2_SO_4_ (TMS), was designed and prepared for thermal energy storage (TES) to address the issues of the narrow temperature range and low specific heat of solar salt molten salt. The thermo-physical properties of TMS-2, such as melting point, decomposition temperature, fusion enthalpy, density, viscosity, specific heat capacity and volumetric thermal energy storage capacity (E_TES_), were determined. Furthermore, a comparison of the thermo-physical properties between commercial solar salt and TMS-2 was carried out. TMS-2 had a melting point 6.5 °C lower and a decomposition temperature 38.93 °C higher than those of solar salt. The use temperature range of TMS molten salt was 45.43 °C larger than that of solar salt, which had been widened about 13.17%. Within the testing temperature range, the average specific heat capacity of TMS-2 (1.69 J·K^−1^·g^−1^) was 9.03% higher than that of solar salt (1.55 J·K^−1^·g^−1^). TMS-2 also showed higher density, slightly higher viscosity and higher E_TES_. XRD, FTIR and Raman spectra SEM showed that the composition and structure of the synthesized new molten salt were different, which explained the specific heat capacity increasing. Molecular dynamic (MD) simulation was performed to explore the different macroscopic properties of solar salt and TMS at the molecular level. The MD simulation results suggested that cation–cation and cation–anion interactions became weaker as the temperature increased and the randomness of molecular motion increased, which revealed that the interaction between the cation cluster and anion cluster became loose. The stronger interaction between Na-SO_4_ cation–anion clusters indicated that TMS-2 molten salt had a higher specific heat capacity than solar salt. The result of the thermal stability analysis indicated that the weight losses of solar salt and TMS-2 at 550 °C were only 27% and 53%, respectively. Both the simulation and experimental study indicated that TMS-2 is a promising candidate fluid for solar power generation systems.

## 1. Introduction

Renewable energy has gained increasing attention in recent years due to rising global energy demand, climate change and serious global environmental pollution [1,2,3,4,5]. Solar energy will play an important role in future sustainable energy systems. However, because of its intermittent and unstable supply, it is necessary to develop low-cost TES materials and efficient thermal storage technology. Concentrating solar power (CSP) containing TES materials has become a significant environmentally friendly technology for harvesting the energy of the sun to generate electricity [6,7,8,9,10]. As is well known, molten salt is currently the most widespread and most mature TES materials in CSP systems due to its good thermal properties (wide working temperature range, moderate density, low viscosity, high thermal conductivity and large heat capacity), low cost and non-toxicity [11,12,13,14]. To date, many molten salts such as nitrate, carbonate, chloride, sulfate and fluoride salts have been studied as TES depending on the solar collector type and required operating temperature range [15,16,17,18,19,20]. Compared with other salts, nitrate salts have become commercially available for CSP plants due to their low melting point and low cost, which shows that molten salts offer practical engineering applications and very broad application scenarios [21,22,23,24].

The main nitrate salts, such as solar salt (60wt% NaNO_3_ + 40wt% KNO_3_), Hitec salt (53%wt KNO_3_ + 40wt% NaNO_2_ + 7wt% NaNO_3_) and Hitec XL salt (7% NaNO_3_+ 45% KNO_3_ + 48% Ca(NO_3_)_2_) [25,26,27], are commonly used today in CSP. However, solar salt has a low operating temperature (around 565 °C) [12] and low specific heat (1.47 J·K^−1^·g^−1^) [28]; Hitec salt also has a low specific heat (1.42 J·K^−1^·g^−1^) [29]; and Hitec XL salt has a low operating temperature (524.34 °C), small specific heat (1.312 J·K^−1^·g^−1^) and high viscosity (14cP, 300 °C) [27,30]. A narrow working temperature and small specific heat capacity would lead to potentially lower heat-to-electric conversion efficiencies and increase the scale of thermal storage systems. In recent years, there have been many attempts to improve the working temperature upper limit and specific heat capacity based on nitrates. Some new mixed molten salts based on nitrate have been designed and prepared for improving the working temperature upper limit. 

Han et al. [31] developed quaternary nitrate–nitrite mixed salt, NaNO_3_-KNO_3_-NaNO_2_-Ca(NO_3_)_2_, and found that the decomposition temperature was about 629.9 °C. However, adding Ca(NO_3_)_2_ gives rise to viscosity enhancement. Fernández et al. [27] added LiNO_3_ into solar salt prepared with ternary salt and found that the decomposition temperature increased to 586.32 °C, while LiNO_3_ was expensive. Dunlop et al. [32] and Castro-Quijada et al. [33] found that the addition of a variety of chlorides into Hitec salt or solar salt had the ability to increase the decomposition temperature of molten salt. Sang et al. designed and prepared a new KNO_2_-KNO_3_-K_2_CO_3_ molten salt [6]; it was found that the decomposition temperature could reach 750.1 °C, but its melting point was up to 348.7 °C. Sang et al. [34] also designed and prepared NaNO_3_-KNO_3_-KCl/K_2_CO_3_ molten salt, the melting points of which were 206.12 °C and 207.24 °C, respectively. Moreover, their decomposition temperatures were increased to more than 640 °C. The specific heat capacities of NaNO_3_-KNO_3_-KCl/K_2_CO_3_ in the liquid region were averaged at 1.5298 J/(g·K) and 1.3724 J/(g·K), respectively. From previous research work on nitrate molten salts, most work are based on chlorides and carbonate, while chlorides may increase the corrosion of molten salt on materials such as stainless steel [35,36], and carbonate reduces the specific heat capacity. Robelin et al. [37] reported calculated temperatures of the ternary invariant points on the liquidus of the (NaNO_3_ + KNO_3_ + Na_2_SO_4_ + K_2_SO_4_) system. It was found that the lowest ternary invariant point on the liquidus in the (NaNO_3_ + KNO_3_ + Na_2_SO_4_ + K_2_SO_4_) system was about 217 °C, which was close to melting point of solar molten salt. However, regarding the preparation of a new molten salt based on solar salt containing sulfate, knowledge of its thermo-physical properties and explanations about its structure are still insufficient. 

In this study, we designed and prepared a novel ternary molten nitrate salt of TMS. Meanwhile, experimental and MD simulations were carried out to reveal the structure, morphology and thermophysical properties of TMS. A comparison between the performance of TMS and commercial solar salt was carried out to confirm the feasibility of the ternary salt TMS to be a potential TES material for CSP. 

## 2. Results and Discussion

### 2.1. Thermal Properties

**Figure 1:**
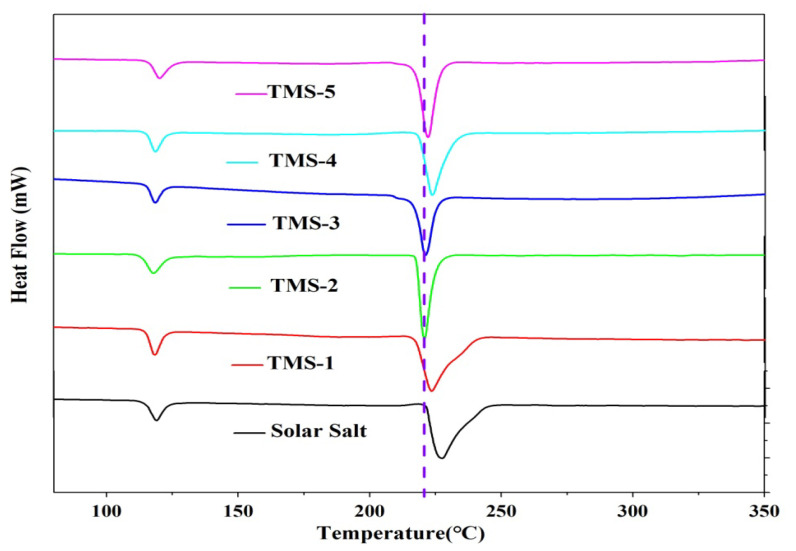
DSC curves of solar salt and TMS system.

**Figure 2:**
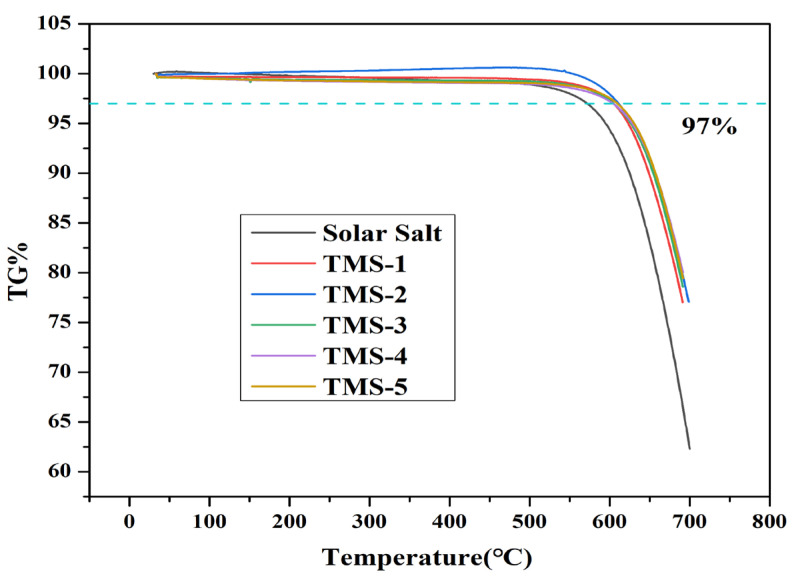
TG curves of solar salt and TMS system.

The DSC and TG experimental results for solar salt and TMS are presented in Figure 1 and Figure 2 and Table 1, respectively. The smaller sharp endothermic peak of solar salt and TMS at 117–120 °C represents a solid–solid phase transition of KNO_3_, while the peak at 220–228 °C represents a solid–liquid phase change. The melting point of solar salt was 227.47 °C, which was consistent with the results reported in the literature [38,39,40]. The melting point of TMS ranged from 220.97 °C to 223.68 °C. The lowest melting point of TMS was 220.97 °C, with a composition of NaNO_3_ (42.01%)-KNO_3_ (56.30%)-Na_2_SO_4_ (1.69%), which was consistent with the results reported in the literature [34]. TMS-2 had a melting point 6.5 °C lower than that of solar salt. Furthermore, the TG curves suggested that the decomposition temperatures of solar salt and the TMS system were different. The decomposition temperature of solar salt was 572.32 °C, which was consistent with the results reported in the literature [38,39,40]. From Figure 2, it can be seen that the decomposition temperatures of the TMS series of molten salt were within the range of 604.01 °C–611.25 °C, with a sodium sulfate content of 1–5%, with a small change. The highest decomposition temperature was 611.25 °C, which was 38.93 °C higher than that of solar salt. The use temperature range of TMS molten salt was 45.43 °C larger than that of solar salt, which had been widened about 13.17%. This was because the decomposition temperature of sodium sulfate was 884 °C, so the TMS decomposition temperature significantly increased while significantly improving the thermal storage performance and photothermal conversion efficiency of TMS. The latent heat of the phase transition of ternary interactive TMS molten salt increased from 94.86 J∙g^−1^ to 117.5 J∙g^−1^, with little change compared to solar salt. In short, the addition of sodium sulfate broadened the use of the temperature zone and reduced the cost to a certain extent. These results show that TMS was more stable and had a larger range of working temperature than solar salt.

### 2.2. Thermo-Physical Properties

#### 2.2.1. Density

Density is one of the important thermophysical parameters of TES materials. On the one hand, the level of density is related to the volume of the entire heat storage system and the design cost of the heat storage system. On the other hand, density and specific heat capacity together determine the energy storage capacity of molten salt materials, which affects the efficiency of energy storage systems [41]. 

**Figure 3:**
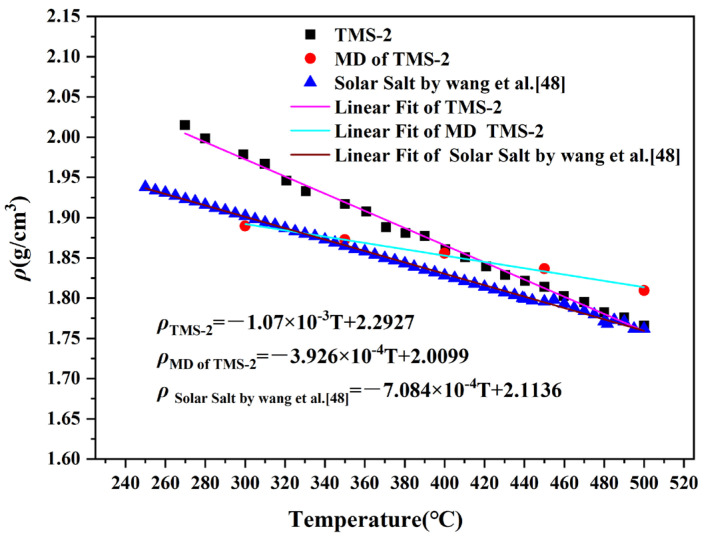
Density curves of solar salt and TMS-2.

**Figure 4:**
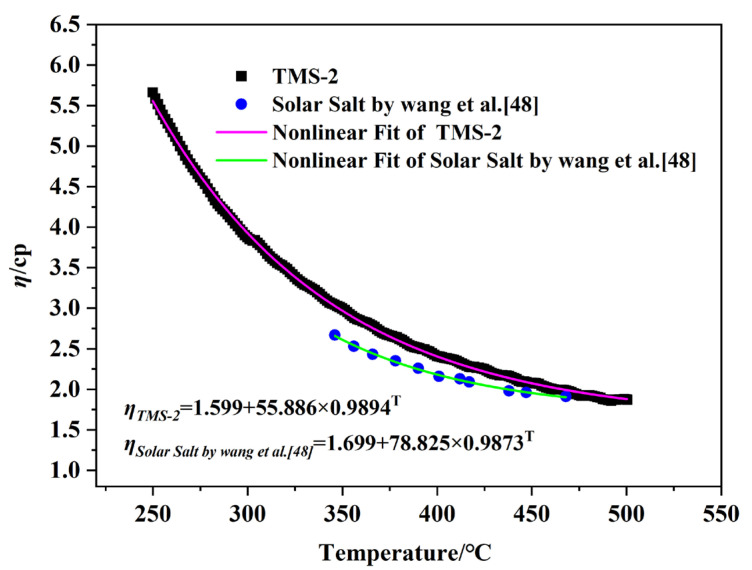
Viscosity comparison of solar salt and TMS-2 as a function of temperature.

**Figure 5:**
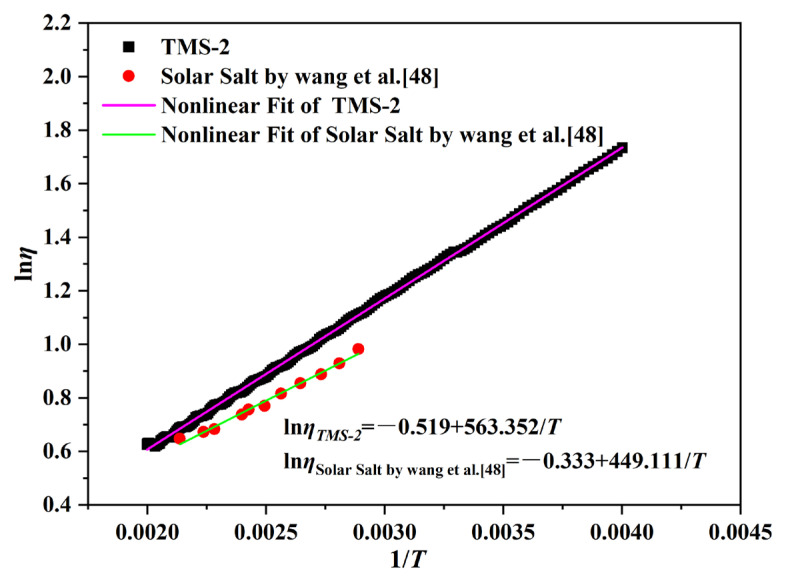
Activation energies for viscous flow (*E_η_*) as a function of temperature.

In the molten state, the density of molten salt varies greatly depending on temperature, and usually has a linear relationship with temperature, which can be expressed as ρ = A-BT [42,43]. The density of TMS-2 increased with temperature from 260 °C to 500 °C, as shown in Figure 3. Through regression analysis, the A and B constants of TMS-2 can be determined. To speculate on the density of molten salt at unknown temperatures, MD was used to simulate the densities at 300 °C, 350 °C, 400 °C, 450 °C and 500 °C. It can be seen from Figure 4 and Figure 5 that the error between the simulation results and the experimental values was between 0.31% and 4.5%, which was smaller compared to the literature reports [44]. This indicated that the simulation data can also be used to speculate on the density of unknown temperatures while verifying the accuracy of the calculation simulation model. Figure 3 also shows that the density of TMS-2 decreased linearly with temperature in the molten state over the testing temperature range. From Figure 3, it can be seen that the simulated density was less than the experimental value when the temperature was less than 400 °C, and greater than the experimental value when the temperature was greater than 400 °C. Compared with the solar salt [44,45], the experimental density of TMS-2 was slightly higher than the density of solar salt. A higher density can also reduce the volume of the molten salt storage system and its costs.

#### 2.2.2. Viscosity

The flow of liquid molten salt in pipelines (viscosity) is crucial for the safety and efficiency of CSP, which is an important factor affecting the cost of molten salt pumps in the system. Low-viscosity systems can reduce energy consumption during the pumping process by reducing friction, thereby reducing costs [46]. The viscosity curves of TMS-2 and solar salt as a function of temperature are shown in Figure 4. It can be clearly seen that the viscosity of TMS-2 and solar salt decreased with increasing temperature. This was because as the temperature increased, the increasing energy reduced the Coulomb force between ions, resulting in an increase in liquid fluidity and a decrease in viscosity. In the studied temperature range, the viscosity of solar salt was slightly lower than that of TMS-2, indicating that when used as a molten salt energy storage material, the energy consumption of TMS-2 increased slightly.

The change in viscosity (*η*) with temperature is given by Equation (1) [47]:

(1) $$\ln\eta =\ln\eta_{0}+\frac{E_{\eta }}{RT}$$

in which *η_0_* is a constant, $E_{\eta }$ is the energy for the activation of viscous flow, and R is the gas constant. Figure 5 shows that all the data in Figure 5 fit well with Equation (1). It is noted that the viscosity of TMS-2 and solar salt was inversely linked to their $E_{\eta }$. The values obtained for $E_{\eta }$ help with the design of low-viscosity molten salt.

#### 2.2.3. Specific Heat Capacity

**Figure 6:**
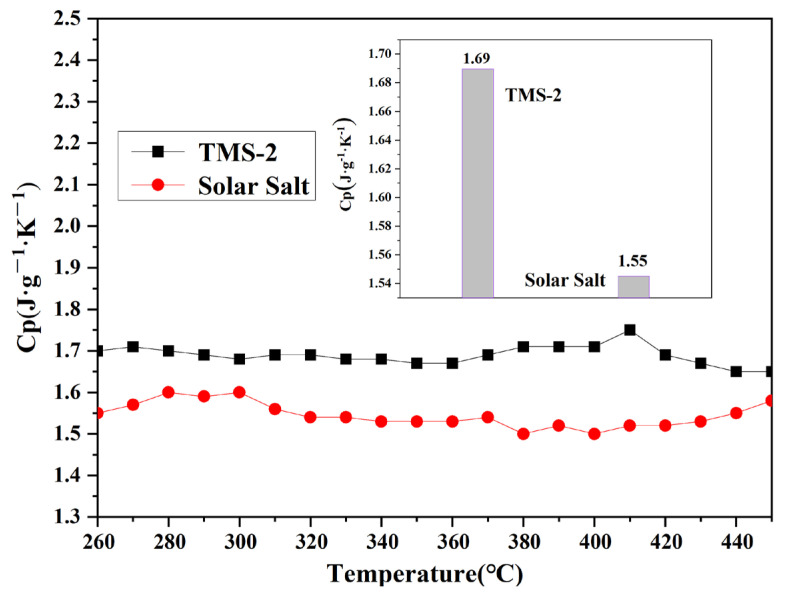
Specific heat capacity comparison of solar salt and TMS-2.

The specific heat capacity represents the amount of heat absorbed per unit temperature per unit mass of molten salt, and is an important parameter characterizing the thermal storage performance of molten salt energy storage materials. The specific heat capacity of TMS-2 molten salt and solar molten salt in the molten state varied with temperature, as shown in Figure 6. It is noted that the specific heat capacity of TMS-2 and solar salt was not significantly related to temperature changes. Within the testing temperature range, the average specific heat capacities of TMS-2 and solar salt were 1.69 J·K^−1^·g^−1^ and 1.55 J·K^−1^·g^−1^, respectively. The specific heat capacity of TMS-2 was 9.03% higher than that of solar salt. This was because of the differences in the composition and morphology of TMS-2 and solar salt. The above results indicate that TMS-2 molten salt had certain advantages in the application of CSP in terms of specific heat capacity compared with solar salt.

#### 2.2.4. Thermal Conductivity

**Figure 7:**
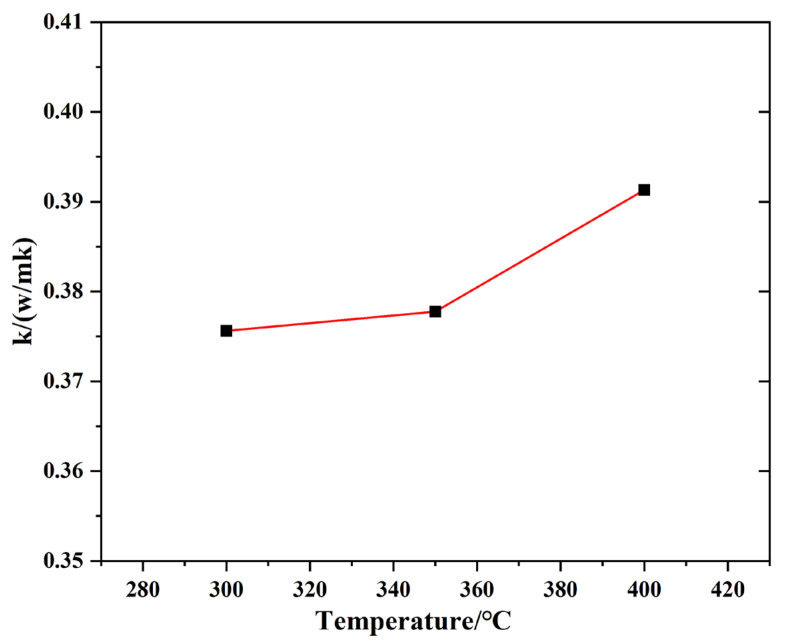
Thermal conductivity of TMS-2 as a function of temperature.

Thermal conductivity reflects the heat transfer conditions of liquid molten salts at high temperature. The thermal conductivity of TMS-2 can be calculated using Equation (2):

k = Cp × ρ × α(2)

where Cp is the specific heat capacity, ρ is the density and α is the thermal coefficient. The thermal conductivity of TMS-2 at 300 °C–400 °C in nitrogen is shown in Figure 7. The thermal conductivity of TMS-2 was higher, similar to solar salt [45].

### 2.3. Thermal Energy Storage Capacity

The thermal energy storage capacity (E_TES_) seriously affects the efficiency and cost of CSP. The thermal energy storage capacity can be calculated using Equation (3):

E_TES_ = Cp × ρ × ΔT (3)

where ρ is the density, Cp is the specific heat capacity and T is the working temperature.

**Figure 8:**
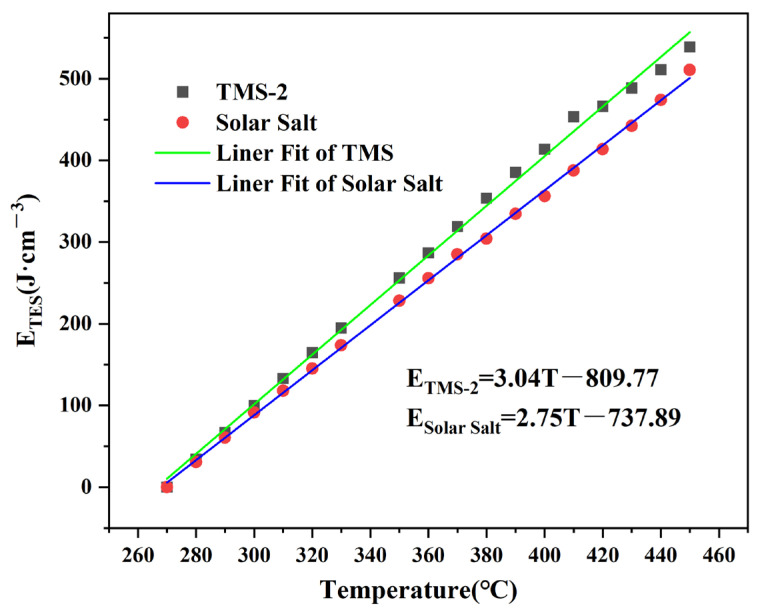
Thermal energy storage (TES) capacity of TMS-2 and solar salt.

The inlet temperature was 50 °C higher than the melting point in order to meet industrial needs. For TMS-2 and solar salt, 270 °C was confirmed as the inlet temperature. Figure 8 shows the E_TES_ of TMS-2 and solar salt in the temperature range of 260–460 °C. The E_TES_ of the two molten salts increased with the increasing temperature. The higher E_TES_ of TMS-2 than that of solar salt indicated that TMS-2 was expected to be the alternative fluid for energy storage.

### 2.4. XRD Analysis

**Figure 9:**
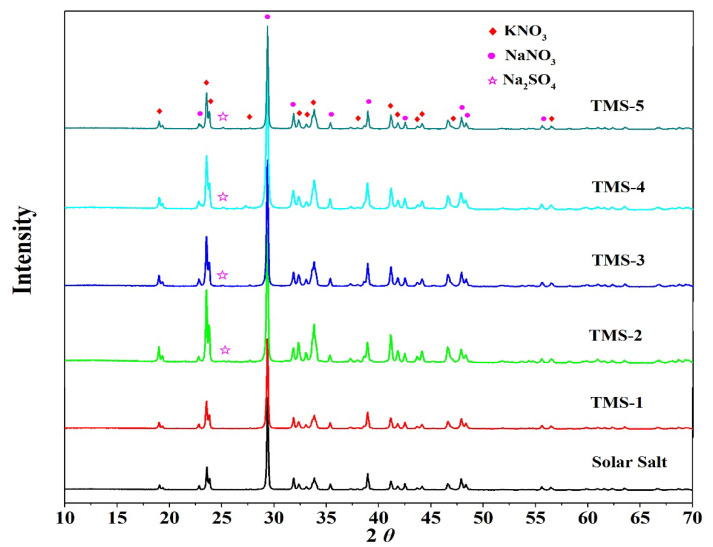
XRD patterns of solar salt and TMS system.

To confirm the composition of the new molten salt, the TMS series of molten salt and solar salt were characterized by XRD. Figure 9 shows the XRD spectra of the TMS series and solar salt. It is noted that solar salt was formed of potassium nitrate and sodium nitrate and the TMS system consisted of potassium nitrate, sodium nitrate and sodium sulfate. In the TMS series of molten salt, a characteristic peak of sodium sulfate appeared at 25° from TMS-2 to TMS-5. As the sodium sulfate content increased from 1.69 to 5%, the intensity of the characteristic peak increased. Due to the sodium sulfate content being lower than the detection limit of XRD, no sodium sulfate appeared in TMS-1. The XRD data showed that the composition of the synthesized new molten salt was consistent with the design synthesis.

### 2.5. FTIR Characterization

**Figure 10:**
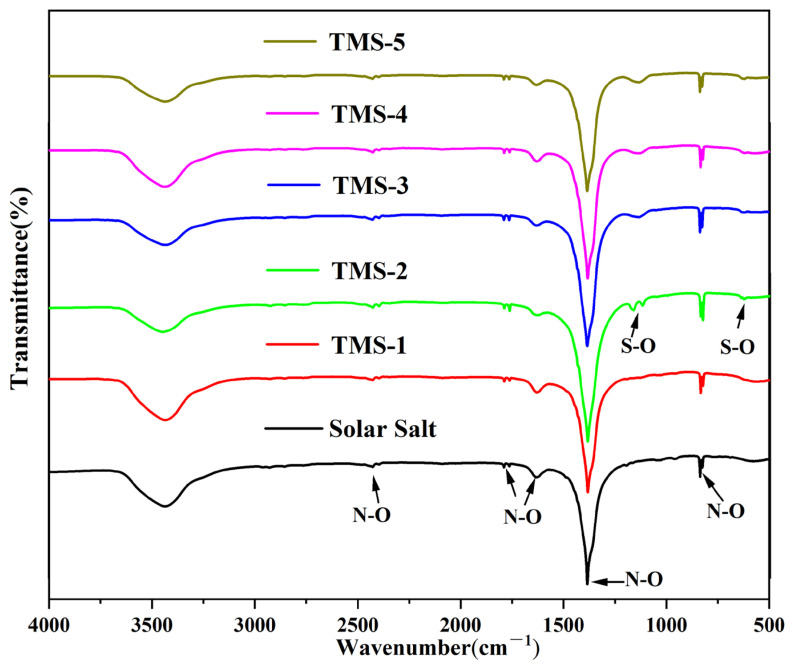
FTIR of solar salt and TMS system.

The FTIR spectra of solar salt and the TMS series are shown in Figure 10. It is seen that the characteristic absorption peak near 833 cm^−1^ corresponded to the N-O bending mode. The characteristic wide peak at 1382 cm^−1^ corresponded to the N-O asymmetric stretching mode. The absorption band at 1628 cm^−1^ was because of the 2-fold bending peak of N-O. The two weak bands at around 1790 cm^−1^ corresponded to the symmetric stretching and asymmetric planar bending of N-O. The weak bands at around 2484 cm^−1^ corresponded to the symmetric and asymmetric stretching modes of N-O [48]. When the content of sodium sulfate was greater than 1.69% (TMS-2), the characteristic peak of 620 cm^−1^ in the infrared spectrum belonged to the S-O symmetric stretching mode, and the characteristic wide peak at 1130 cm^−1^ belonged to the S-O asymmetric stretching mode [49]. Due to the low content of sodium sulfate in TMS-1, no characteristic peak of S-O was detected. The infrared spectrum results showed that the composition of the synthesized new molten salt was consistent with the design synthesis, which validated the XRD results.

### 2.6. Raman Spectra

**Figure 11:**
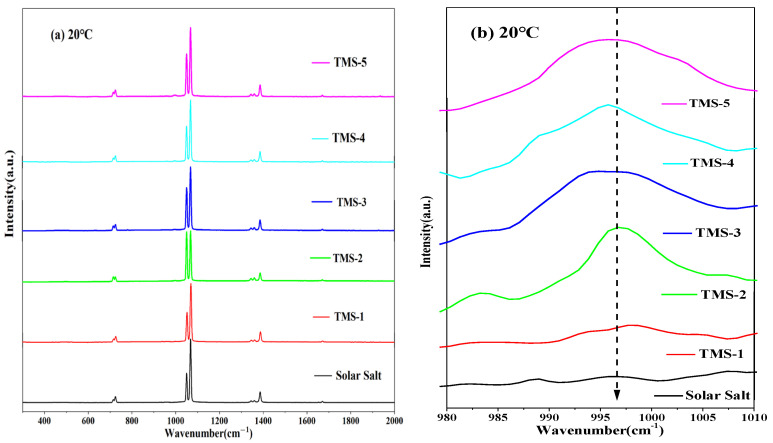
Raman diagrams of solar salt and TMS series of molten salt at 20 °C ((**a**) 300 cm^−^^1^ to 2000 cm^−^^1^; (**b**) 980 cm^−^^1^ to 1010 cm^−^^1^).

**Figure 12:**
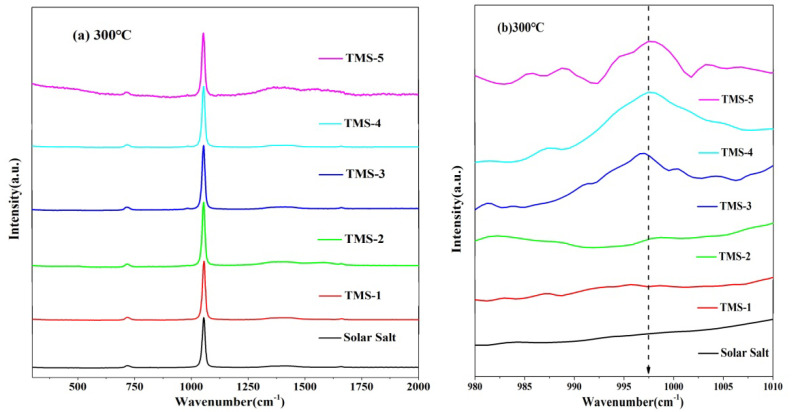
Raman diagrams of solar salt and TMS of series molten salt at 300 °C ((**a**) 300 cm^−1^ to 2000 cm^−1^; (**b**) 980 cm^−1^ to 1010 cm^−^^1^).

The structural variation information of the corresponding substances can be characterized by the Raman spectra. Figure 11 and Figure 12 show the Raman spectra for the solid state (20 °C) and molten state (300 °C) of solar salt and TMS series of molten salt from 300 cm^−1^ to 2000 cm^−1^, respectively. It is clearly observed that the bands at about 716 cm^−1^, 1050 cm^−1^ and 1358 cm^−1^ at 20 °C in the solid state of solar salt and TMS series of molten salts were attributed to the vibrations of NO_3_^−^ in KNO_3_.Furthermore, 727 cm^−1^, 1070 cm^−1^ and 1386 cm^−1^ corresponded to NO_3_^−^ in NaNO_3_. Compared with 20 °C, at 300 °C, the characteristic peaks of NO_3_^−^ of the stretching vibration peaks in KNO_3_ and NaNO_3_ were changed into one peak at about 730 cm^−1^ and 1050 cm^−1^ because of the inhomogeneity and randomness of the ion cluster microstructure with increasing temperature [50], while weaker peak shapes at about 130–1440 cm^−1^ were not observed. This also illustrated that the arrangement of the cations around NO_3_^−^ was very uniform when the two systems were in a solid state [51,52]. It can be seen from Figure 11b that the weak characteristic peak at 995–1000 cm^−1^ was because of SO_4_^2−^ when the mass fraction of sodium sulfate was greater than or equal to 1.69% at 20 °C. It was concluded that a new molten salt containing sodium sulfate was synthesized. Due to the low content of sodium sulfate in TMS-1, no characteristic peak of S-O was detected, which was consistent with the conclusion of XRD and FTIR. However, Figure 11b shows a weak characteristic peak at 995–1000 cm^−1^ belonging to the Raman peaks of SO_4_^2−^ when the mass fraction of sodium sulfate was greater than or equal to 2% at 300 °C in the molten state of TMS. This was because of an increase in the randomness of the ion cluster microstructure while temperature increased. It is concluded that the stronger interaction between Na^+^ and SO_4_^2−^ in TMS-2 contributed to the higher specific heat.

### 2.7. SEM Characterization

**Figure 13:**
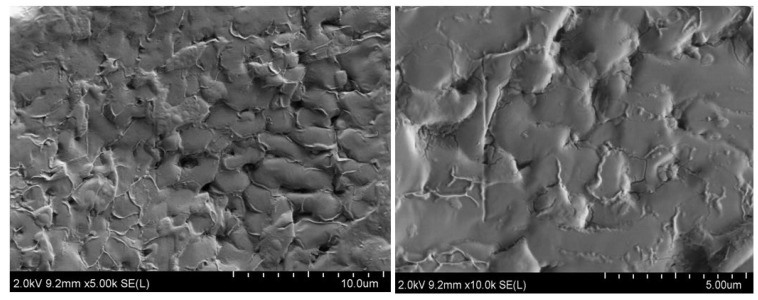
SEM images of TMS-2.

Figure 13 shows a SEM image of the surface of the TMS-2 molten salt crystal. In Figure 13, it can be clearly observed that the surface of TMS-2 molten salt was uniformly distributed with strip-like protrusions compared to the smooth surface morphology of solar salt reported in the literature [30]. The particle size of TMS-2 molten salt was larger than that of solar salt. Larger particles may be able to increase specific heat capacity.

**Figure 14:**
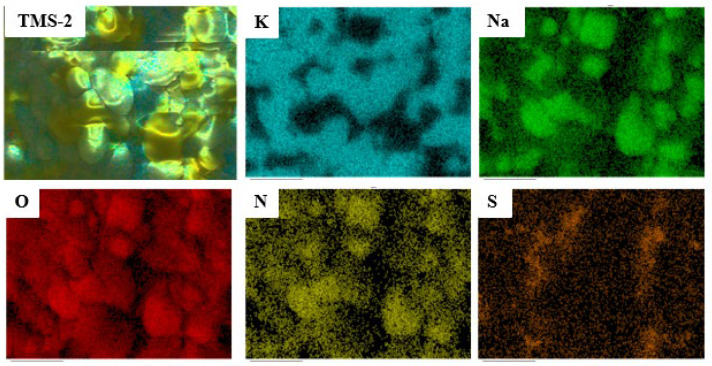
Mapping of TMS-2.

The EDS mapping spectrum of TMS-2 molten salt is presented in Figure 14. The position and distribution of each element can be clearly observed from Figure 14. The content of the Na element in TMS-2 molten salt was lower than that of the K element, which was consistent with the designed molten salt ratio. Figure 14 also shows that the K and Na elements in TMS-2 molten salt exhibited a certain complementary distribution. The formation of this complementary distribution structure may be related to the strong repulsion between K^+^ and Na^+^. The uniform distribution of sulfate ions within the Na element distribution area indicated a stronger binding force between sulfate ions and sodium. The strong repulsion between K^+^ and Na^+^ and stronger binding force between sulfate ions and sodium may have resulted in higher specific heat of TMS-2 molten salt than that of solar salt.

### 2.8. Molecular-Scale Analysis

#### 2.8.1. RDF

In order to further explain the reasons for the different thermal parameters such as the density, viscosity and specific heat capacity of TMS-2 molten salt, the MD was used to calculate the RDF, coordination number and ADF of TMS-2 molten salt at 300 °C, 350 °C, 400 °C, 450 °C and 500 °C. The differences in thermal properties were investigated at the molecular microstructure level.

**Figure 15:**
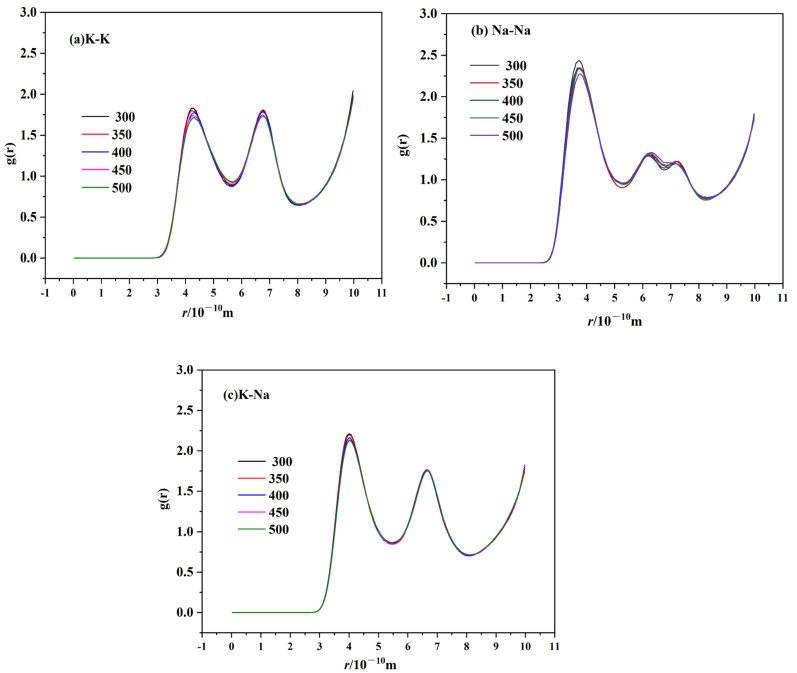
RDF of cation–cation ion pairs of TMS-2 ((**a**) K-K; (**b**) Na-Na; (**c**) K-Na).

**Figure 16:**
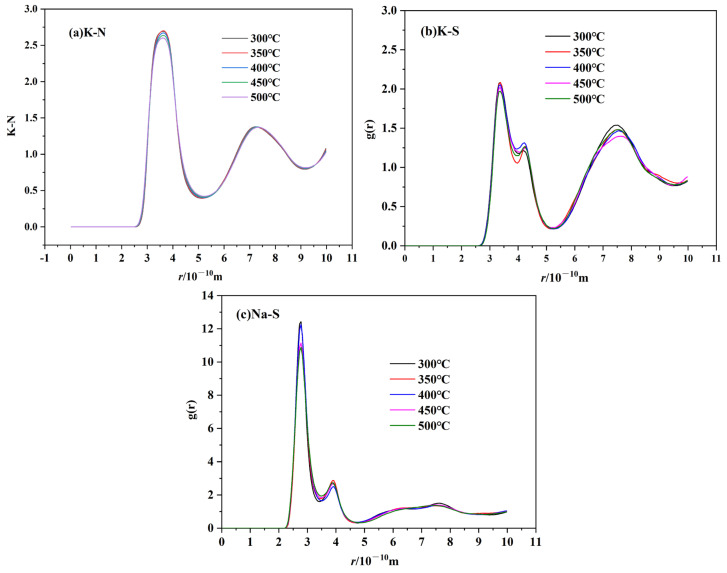
RDF of cation–anion ion pairs of TMS-2 ((**a**) K-NO_3_^−^; (**b**) Na-NO_3_^−^; (**c**) K-SO_4_^2−^.

RDF can be used to describe the basic structure of molten salts. The RDFs of cation–anion and cation–cation for TMS-2 molten salt at 300 °C, 350 °C, 400 °C, 450 °C and 500 °C are shown in Figure 15 and Figure 16. All RDFs exhibited the following same features: a relatively high first peak followed by some lower peaks, and the peaks approaching unity at a larger distance. It is noted that the arrangement of ions in the molten state of TMS-2 exhibited long-range disorder and short-range order. It can be seen that the first cation–cation peak was wider and its peak valley was higher than that of cation–anion, which was because a strong coordination structure was formed between cations and anions. Meanwhile, the cation–anion curves were opposite to the corresponding ones of cation–cation, indicating that cations and anions were regularly interlaced.

Figure 16 shows that the height of the first cation–cation peak decreased as the temperature increased, and the peak position shifted to the right. While it can be seen from Figure 16 for cation–anion that the height of the first cation–anion peak decreased as the temperature increased, there was no significant change in the overall morphology of RDF. This was because the cation–cation and cation–anion interactions became weaker as the temperature increased and the randomness of molecular motion increased, which suggested that the interaction between the anion cluster and cation cluster became loose. The loose arrangement of ion clusters helped to reduce viscosity and density as the temperature increased. The distance between cations increased as the temperature increased, which caused the peak position to shift to the right. However, the Coulombic attraction between cations and anions was stronger, and the temperature had no significant effect on the distance between cations and anions. For cation–anion pairs, as shown in Figure 16 it is obvious that the peak height of Na-SO_4_ was significantly higher than that of K-SO_4_. This may be due to the presence of sodium sulfate in the molten salt, which was consistent with the XRD, infrared and Raman results. Figure 16 also shows that the peak height of Na-SO_4_ was significantly higher than that of K-N and Na-N, which indicated a stronger interaction between Na-SO_4_ cation and anion clusters. Therefore, TMS-2 molten salt had a higher specific heat capacity than solar salt.

In Figure 15, it is obvious that the second peak height of cation–cation pairs of TMS-2 molten salt was stronger than that of solar salt [53]. Classically, the second peak becoming stronger indicated that the ions grew in regularity [54]. So, the density and viscosity of TMS-2 were higher than those of solar salt because the distance of the ion clusters of TMS-2 was smaller.

#### 2.8.2. Coordination Number

The coordination number (N_αβ_) can illustrate the structure of TMS-2. It is described using Equation (4), where ρ_β_ is the mean number of β-type ions lying in a sphere cavity of radius r min centered on an α-type ion, and r min is the position of the first peak valley of the RDF.

(4) $$N_{\alpha \beta }\left(r\right)=4\pi \rho_{\beta }\int_{0}^{r_{min}}g_{\alpha \beta }\left(r\right)r^{2}dr$$

The cation–anion coordination numbers of TMS-2 are shown in Table 2. It is obvious that the coordination number of all ion pairs decreased as the temperature increased, indicating that ion clusters became loose at the microscopic level as the temperature increased. The coordination number of Na-NO_3_ was greater than that of K-NO_3_, and the peak of Na-NO_3_ in the radial distribution function was higher than that of K-NO_3_. This indicated that there were more anions in the first coordination shell of Na than K, and Na-NO_3_ bound more tightly than K-NO_3_. The coordination number of K-SO_4_ was much smaller than that of Na-SO_4_, which illustrated that sulfate ions in TMS-2 molten salt mainly existed in the form of sodium sulfate.

#### 2.8.3. Angular Distribution Functions (ADF)

**Figure 17:**
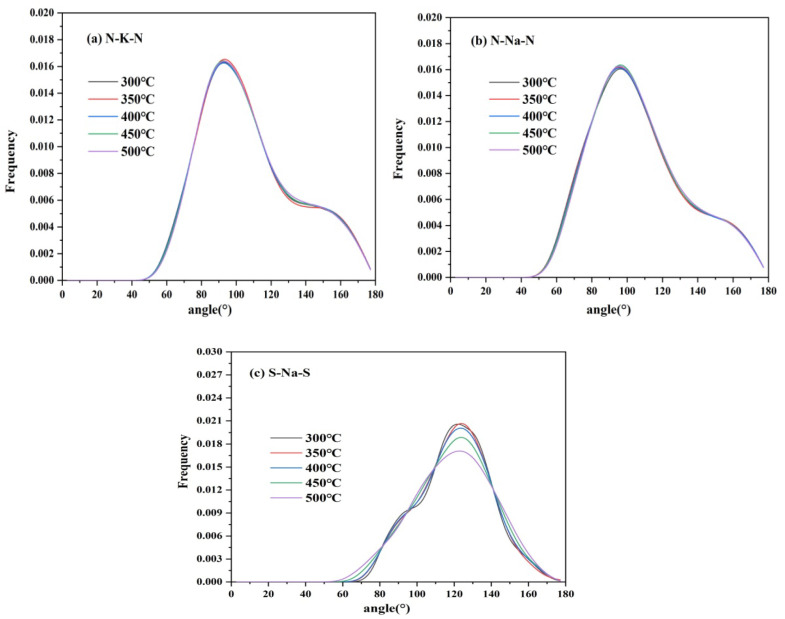
ADF of cation–anion ion pairs of TMS-2 ((**a**) N-K-N; (**b**) N-Na-N; (**c**) S-Na-S).

Figure 17 shows the ADF of cation–anion pairs of TMS-2. It is noted that the bond angles of TMS-2 were distributed within the range of 30–180°. The bond angles of N-K-N and N-Na-N were relatively concentrated and slightly higher than 90°, indicating that the coordination bond orientation was regular and tended to form a loose octahedral coordination structure. The peak of N-K-N was higher than that of N-Na-N and closer to 90°, indicating that the octahedral coordination configuration of N-Na-N was more significant than that of N-K-N. The bond angle of S-Na-S was mainly concentrated around 120°, indicating a regular orientation of coordination bonds and a tendency to form loose tetrahedral coordination structures. The peak intensity of the S-Na-S bond angle became weak as the temperature increased, indicating that as the temperature increased, the interaction between ions weakened, the randomness of molecular motion increased, and the micro clusters of molecules became somewhat loose.

### 2.9. Thermal Stability

**Figure 18:**
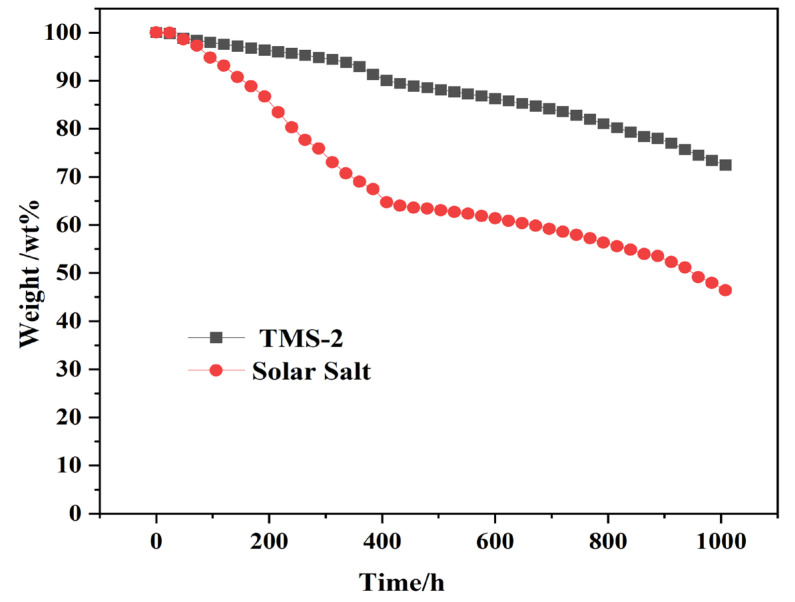
The thermal stability of TMS-2 at 550 °C.

The upper limit of the use temperature of molten salt is limited by its stability at high temperatures. Generally, the upper limit of the use temperature is about 50 °C lower than the decomposition temperature. The long-term stability of TMS-2 molten salt and solar molten salt at 550 °C was investigated, as shown in Figure 18. It can be seen that at 550 °C, the weight loss of solar salt was significantly higher than that of TMS-2 molten salt. The weight loss further increased as time went on. After 1008 h, the weight loss of solar salt was as high as 53%, while the weight loss of TMS-2 was only about 27%. This indicated that TMS-2 has better stability at 550 °C.

## 3. Materials and Methods

### 3.1. Materials and Synthesis

KNO_3_, NaNO_3_ and Na_2_SO_4_ used in this experiment were analytical grade. They were purchased from Sinopharm Chemical Reagent Beijing Co., Ltd. (Beijing, China).

**Figure 19:**
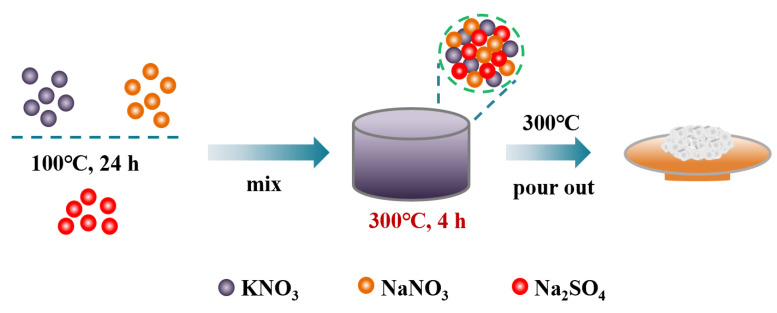
Preparation process of solar salt and TMS system molten salt.

NaNO_3_, KNO_3_ and Na_2_SO_4_ were dried in a constant-temperature air-drying oven at 80 °C for 24 h to remove moisture from raw materials. In this study, molten salts were synthesized by the static melting method. Briefly, 20 g binary salt of 60wt% NaNO_3_-40wt% KNO_3_ and ternary eutectic salt of TMS with different proportions, calculated according to the phase diagram in the literature [37], as shown in Table 3, were mixed into a 50 mL crucible. Then, the mixture salts were heated up to 300 °C with a heating rate of 5 °C/min in a muffle furnace and were kept at 300 °C for 4 h. Finally, the synthesized binary and ternary molten salts were poured out at 300 °C and cooled to room temperature naturally, and then crushed into powder, sealed and kept in desiccators. The preparation process is shown in Figure 19.

### 3.2. Characterization of Thermal Properties

The melting point, latent heat of phase change and decomposition temperature are the three key thermophysical parameters of energy storage media. The melting point determines the low-limit usage temperature of the energy storage medium. A low melting point can reduce the heating energy and time of the salt melting system and effectively prevent the occurrence of the “freezing blockage” phenomenon, which reduces the operating cost of the CSP system. The decomposition temperature determines the upper limit of the usage temperature of the energy storage medium. The melting point and decomposition temperature together determine the range of the usage temperature of the energy storage medium [55,56,57].

The melting point, weight loss and fusion enthalpy of the solar salt and TMS system molten salt were measured by TG-DSC (METTLER TOLEDO, TGA/DSC3+). The measurements were carried out at a temperature range of 30–700 °C with a heating rate of 10 °C/min. The blowing gas was nitrogen with a blowing flow rate of 50 mL/min. The crucible used for measurement was a 100 μL platinum crucible with a perforated cover. The measurement error of temperature was within ±0.005 °C and the accuracy of the calorimeter was within ±1%. The specific heat capacity was also measured by TG-DSC, following the standard of ASTM E1269 [58,59]. Firstly, the DSC values of the empty aluminum crucible, molten salts and standard sapphire were measured. The crucible used was an aluminum crucible with a cover, and the quality of the sample needed to be within 0.5 mg of the mass difference with the sapphire standard sample. The measurements were carried out at a temperature range of 30 °C–400 °C with a heating rate of 10 °C/min. The blowing gas was nitrogen with a flow rate of 50 mL/min.

The specific heat was calculated through the following Equation (5):

(5) $$\frac{{\mathrm{DSC}}_{sample}-{\mathrm{DSC}}_{\mathrm{baseline}}}{{\mathrm{DSC}}_{\mathrm{sapphire}}-{\mathrm{DSC}}_{\mathrm{baseline}}}=\frac{{\mathrm{Cp}}_{sample}\cdot M_{s\mathrm{ample}}}{{\mathrm{Cp}}_{sapphire}\cdot M_{s\mathrm{apphire}}}$$

where m and Cp stand for mass and specific heat capacity, respectively.

The density was determined by a high-temperature t density meter (MSP-2, Shanghai YUZHI technology, Shanghai, China) followed the Archimedes method. The experiments were carried out over a range of 250–500 °C with a cooling rate of 1 °C/min in air.

The viscosity of solar salt and TMS was determined by a modular compact rheometer (MCR) (MCR 502, Graz, Austria) that operated by means of rotary oscillation [60]. The viscosity was measured by a steady-state test at a shear rate of 50 s^−1^ over a range of 250–500 °C with a cooling rate of 2 °C/min. The amounts of samples required were about 12–15 g.

### 3.3. Material Characterization

XRD (D8 Discover, Bruker, Germany) was applied to identify the solid phase structure of solar salt and TMS. Diffraction patterns were obtained by using a diffractometer (Cu K α radiation (λ = 1.5406 Å)). They were analyzed in 2θ ranging from 5° to 80° with a 0.017° step size per step under a generator voltage of 40 kV and a tube current of 30 mA. The morphology of the solar salt and TMS was measured by SEM (JSM-5610LV, JEOL, Japan) in combination with energy-dispersive X-ray spectroscopy mapping (X-MAXN).

FTIR spectroscopy analysis was conducted by a Nicolet Nexus 670 FTIR Spectrophotometer (Thermo Nicolet Corporation, Madison, WI, USA) in solid films using KBr salt tablets in a range of 500–4000 cm^−1^.

Raman spectra were determined at 20 °C and 300 °C by a Raman spectrometer (DXR, Thermo Fisher Scientific, USA). The samples were ground into powder and carefully placed in the Raman microscope. The spectra were collected in a range of 100–3000 cm^−1^ in an atmosphere of N2 with a Raman shift of 1.9285 cm. Each spectrum came from a single scan and the total collection time was 10 s. However, due to the very small spot size, several spots of each mixture were measured in order to avoid the measurement of impurities as well as single components of the different salt formulations.

### 3.4. MD Simulations

MD simulations were employed to describe the intrinsic relationship between thermo-properties and structure using the open-source molecular simulation package LAMMPS.

The Lennard-Jones potential function was applied to describe the Coulomb and van der Waals interactions between simulated system molecules in this study, as shown in Equation (6):

(6) $$E_{LJ}\left(r_{ij}\right)=4\varepsilon_{ij}\left({\left(\frac{\sigma_{ij}}{r_{ij}}\right)}^{12}-{\left(\frac{\sigma_{ij}}{r_{ij}}\right)}^{6}\right)+\frac{q_{i}q_{j}}{4\pi \varepsilon_{o}\varepsilon_{r}r_{ij}}$$

where *q_i_*, *q_j_* is the atomic charge, r is the distance between atoms, σ is the atomic diameter parameter and *ε* is the van der Waals force field parameter. The potential function parameters are shown in Table 4. For cross terms, the following cross rule was used (Equation (7)).

(7) $$\sigma_{ij}=\frac{1}{2}(\sigma_{ii}{+\sigma }_{jj}),\;\varepsilon_{ij}={\left(\varepsilon_{ii}\varepsilon_{jj}\right)}^{1/2}$$

Harmonic potential was applied to describe the nitrate N-O and sulfate S-O bond use a, as shown in Equation (8).

(8) $$E=K_{r}{\left(r-r_{0}\right)}^{2}$$

(9) $$E=K_{\theta }{\left(\theta -\theta_{0}\right)}^{2}$$

where *r* is the N-O or S-O bond length, *r*_0_ is the equilibrium bond distance and *K_r_* is the force constant. The bond angles of O-N-O and O-S-O were calculated using the harmonic potential, as shown in Equation (9). In the equation, *θ* is the bond angle, *θ*_0_ is the equilibrium degree of the angle and *K_θ_* is the angle parameter. The parameters of intra-molecular pair potential are listed in Table 5.

For the simulation, the case contained 4877 Na^+^, 5630 K^+^, 10,169 NO_3_^−^ and 169 SO_4_^2−^. The initial configuration systems were constructed through the software PACKMOL(v20.4.0) [61], and all the molecules were randomly inserted in a cubic simulation box. The pppm method was employed to calculate long-range electrostatic interactions, and the precision set to 1.0 × 10^−4^.

Firstly, energy minimization was employed to relax the simulation box. Secondly, in order to thoroughly mix the molecules in the system, an isothermal-isobaric (NPT) ensemble with a 1.0 fs time step was employed to optimize the simulation box, where the temperature was set to 1000 K and the pressure was set to 1.0 atm. The temperature and pressure were maintained via a Nose–Hoover thermostat and a Parrinello–Rahman barostat, respectively. This process was set to 5.0 ns. Then, another 5.0 ns was employed to cool the cases, with an NPT ensemble from 1000 K to the target temperature. Thirdly, an equilibrium process was employed in the simulation box, an NPT ensemble with a target temperature, and 1.0 atm was performed for 10.0 ns. Finally, for the analysis production process, an NPT ensemble for radial distribution function (RDF) and angular distribution function (ADF) analysis was performed for 2 ns. Then, an NVT ensemble for viscosity and thermal conductivity analysis was performed for 10 ns. In all the MD simulations, the motion of atoms was described by the classical Newton equation, which was solved using the velocity Verlet algorithm. And all the Molecular Dynamic simulations were performed by using the LAMMPS 2022.07 package [62].

RDF is the most important function for describing the characteristics of fluid structures. RDF also can illustrate the local structure of molten salt, which can be expressed using Equation (10), where ρ_β_ is the number density of β-type ions and N_αβ_(r) is the mean number of β-type ions inside a sphere of radius r centered on an *α*-type ion.

(10) $$g_{\alpha \beta }\left(r\right)=\frac{1}{4\pi \rho_{\beta }r^{2}}[\frac{dN_{\alpha \beta }(r)}{dr}]$$

The bond orientation of TMS-2 molten salt can also be described by ADF [63]. ADF can be described using Equation (11) from a three-body correlation function, where the nearest neighbor anions j and k as end points and the cation ion i as the vertex center.

(11) $$\theta_{ijk}=\langle \cos^{-1}\left(\frac{r_{ij}^{2}-r_{ik}^{2}-r_{jk}^{2}}{2rr_{ij}r_{ik}}\right)\rangle$$

## 4. Conclusions

A novel wide-working-temperature and high-specific-heat-capacity NaNO_3_-KNO_3_-Na_2_SO_4_ (TMS-2) molten salt was synthesized by the high-temperature static melting method, and its composition, structure and thermo-physical properties were shown. Compared with commercial solar salt, the nearly equal melting point and the much higher decomposition temperature of TMS-2 contributed to a larger range of working temperature (enhancement of 38.93 °C). The specific heat capacity of TMS-2 was 9.03% higher than that of solar Salt, which resulted in larger thermal storage capacity. A higher density of TMS-2 could reduce the volume of the molten salt storage system and its costs. XRD, FTIR and Raman spectra showed that the composition of the synthesized new molten salt was consistent with the design synthesis. The Raman spectra also showed that randomness and inhomogeneity of the ion cluster microstructure increased in the liquid of TMS-2 while the temperature increased. The observations from SEM suggested that bigger particles, the formation of a complementary distribution structure, strong repulsion between K^+^ and Na^+^ and a stronger binding force between sulfate ions and sodium may result in a higher specific heat capacity of TMS-2 molten salt than that of solar salt. The results of the MD simulation suggested that the looser arrangement of ion clusters contributed to lower density and viscosity as the temperature increased, while the stronger interaction of Na-SO_4_ contributed to a higher specific heat capacity. The thermal stability indicated that TMS-2 had better stability at 550 °C. In summary, TMS-2 had excellent heat storage, heat transfer performance and working temperature, leading to outstanding application prospects for TMS-2 molten salt in solar power generation systems. However, further research is needed on the corrosion behavior and mechanisms of TMS-2 molten salt to evaluate the interaction of TMS with common structural materials like stainless steels.

## Figures and Tables

**Table 1:** Melting point, decomposition temperature, phase transition enthalpy and working temperature of solar salt and TMS system.

Sample Number	Melting Point/°C	Decomposition Temperature/°C	Phase Transition Enthalpy/J∙g^−1^	Working Temperature/°C
Solar salt	227.47	572.32	113.00	344.85
TMS-1	223.36	605.08	107.81	381.72
TMS-2	220.97	611.25	96.04	390.28
TMS-3	221.11	604.96	94.83	383.85
TMS-4	223.68	604.01	117.59	380.33
TMS-5	222.13	609.63	96.73	387.50

**Table 2:** Coordination numbers of cation–anion ion pairs of TMS-2.

T (°C)	K-N (NO_3_^−^)	Na-N (NO_3_^−^)	K-S (SO_4_^2−^)	Na-S (SO_4_^2−^)
573.15 K	4.12826	5.3302	0.09227	0.20148
623.15 K	4.11057	5.30857	0.09137	0.19793
673.15 K	4.09284	5.27038	0.08898	0.19755
723.15 K	4.05214	5.22777	0.0884	0.1951
773.15 K	4.00338	5.15235	0.08819	0.19475

**Table 3:** Corresponding percentages of NaNO_3_, KNO_3_ and Na_2_SO_4_ in TMS system.

Sample Number	NaNO_3_ (wt%)	KNO_3_ (wt%)	Na_2_SO_4_ (wt%)
Solar salt	60.00	40.00	0.00
TMS-1	54.68	44.32	1.00
TMS-2	42.01	56.30	1.69
TMS-3	41.46	56.04	2.50
TMS-4	54.83	42.17	3.00
TMS-5	45.49	49.51	5.00

**Table 4:** Parameters of inter-molecular pair potential.

Irom	*q*	$\sigma /10^{-10}\;m$	ε/Kcal/mol
Na	1	2.407	0.1531
K	1	3.188	0.0999
N	0.95	3.431	0.0926
O(NO_3_)	−0.65	3.285	0.0799
S	2.2	3.550	0.1250
S(SO_4_)	−1.05	2.960	0.1211

**Table 5:** Parameters of intra-molecular pair potential.

Group	$K_{r}/Kcal/mol\cdot 10^{-20}\;m$	$r_{0}/10^{-10}\;m$	$K_{\theta }/Kcal/mol$	$\theta_{0}/\left({}^{\circ}\right)$
${NO_{3}}^{-}$	525.0	1.2676	105.0	120
${SO_{4}}^{-}$	367.900096	1.78	116.59990	109.47

## Data Availability

The data are contained within the article.

## Symbols and Acronyms

Symbols	
ρ	Density, g/cm^3^
*η*	Viscosity, cp
*E_η_*	Energy for the activation of viscous flow, kJ·mol^−1^
Cp	Specific heat capacity, J·K^−1^·g^−1^
α	Thermal coefficient, -
k	Thermal conductivity, w/mk
E_TES_	Thermal energy storage capacity, J·cm^−3^
N_αβ_	Coordination number
R	Gas constant, J/(K·mol)
Acronyms	
TMS	NaNO_3_-KNO_3_-Na_2_SO_4_
TES	Thermal energy storage
E_TES_	Volumetric thermal energy storage capacity
XRD	X-ray diffraction
FTIR	Fourier transform infrared
SEM	Scanning electron microscope
MD	Molecular dynamics
CSP	Concentrating solar power
DSC	Differential scanning calorimetry
TG	Thermo-gravimetric
RDF	Radial distribution function
ADF	Angular distribution function
